# The Inter- and Intra-Unit Variability of a Low-Cost GPS Data Logger/Receiver to Study Human Outdoor Walking in View of Health and Clinical Studies

**DOI:** 10.1371/journal.pone.0031338

**Published:** 2012-02-20

**Authors:** Pierre Abraham, Bénédicte Noury-Desvaux, Marie Gernigon, Guillaume Mahé, Thomas Sauvaget, Georges Leftheriotis, Alexis Le Faucheur

**Affiliations:** 1 Laboratory of Vascular Investigations and Sports Medicine, University Hospital, Angers, France; 2 CNRS, UMR6214, Inserm U771, Medical School, University of Angers, Angers, France; 3 APCoSS, Institute of Physical Education and Sports Sciences (IFEPSA), UCO 49, Les Ponts de Cé, France; Pennington Biomedical Research Center, United States of America

## Abstract

**Purpose:**

The present study evaluates the intra- and inter-unit variability of the GlobalSat® DG100 GPS data logger/receiver (DG100) when estimating outdoor walking distances and speeds.

**Methods:**

Two experiments were performed using healthy subjects walking on a 400 m outdoor synthetic track. The two experiments consisted of two different outdoor prescribed walking protocols with distances ranging from 50 to 400 m. Experiment 1 examined the intra-unit variability of the DG100 (test-retest reproducibility) when estimating walking distances. Experiment 2 examined the inter-unit variability of four DG100 devices (unit to unit variability) when estimating walking distances and speeds.

**Results:**

The coefficient of variation [95% confidence interval], for the reliability of estimating walking distances, was 2.8 [2.5–3.2] %. The inter-unit variability among the four DG100 units tested ranged from 2.8 [2.5–3.2] % to 3.9 [3.5–4.4] % when estimating distances and from 2.7 [2.4–3.0] % to 3.8 [3.4–4.2] % when estimating speeds.

**Conclusion:**

The present study indicates that the DG100, an economical and convenient GPS data logger/receiver, can be reliably used to study human outdoor walking activities in unobstructed conditions. This device let facilitate the use of GPS in studies of health and disease.

## Introduction

The use of the Global Positioning System (GPS) is an emerging approach in the study of physical activity [1]. GPS has been recently used to study functional limitations in patients with chronic diseases such as multiple sclerosis [2], peripheral artery disease [3], [4] and spine surgery [5], [6]. These studies show that the potential uses of the GPS technique for health and clinical applications are diverse, interesting and promising. Low-cost, lightweight GPS receivers are now commercially available. Such devices can accurately detect walking and resting bouts [7] as well as accurately estimate walking speed and walking distances [7], [8], [9], [10]. They are useful tools for clinical studies that estimate walking speed and maximal walking distance in disabled patients [2], [3], [4], [5], [6]. However, in order to use GPS technology for clinical cohort studies, the technique must be not only accurate but also reliable. This specifically applies to studies that compare two tests of the same patient. In this context, the investigator is expecting that the variability observed is not primarily of technical origin (intra-unit variability). Further, simultaneous use of multiple identical units may be required for clinical cohorts. In that case, it is also important to know the inter-unit variability. Both intra-unit and inter-unit variability should be low. Otherwise, the capability of investigators to observe significant intra-group changes or inter-group differences with GPS-derived parameters may be compromised.

This study examined the GlobalSat® DG100 a lightweight, convenient, low-cost GPS data logger/receiver. This device can also accept an external antenna to improve the reception of satellite signals. As previously shown in a recent study [11], this device produces acceptable accuracy in detecting walking and resting bouts. It also accurately measures walking distances and speeds during detected walking bouts. The purpose of the present study was to investigate the intra- and inter-unit variability of the DG100 when estimating the outdoor walking distances and speeds of healthy subjects.

## Methods

### Population and ethics statement

All experiments were performed using healthy volunteers recruited from the Institute of Physical Education and Sports Sciences in Angers (France). Age, gender and basic anthropometric measures such as height (cm) and body mass (kg) were collected, and body mass index computed, for every subject.

This study was approved by the institutional ethics committee (Comité de Protection des Personnes OUEST II) and registered in the American National Institute of Health database under reference number NCT00485147. For each experiment, experimental procedures were clearly explained and presented to each participant to ensure their understanding and compliance, and their written informed consent was obtained.

### Stated hypotheses

The first hypothesis we tested was that the DG100 has a high level of reliability (low variability) in the measured walking distances resulting from identical prescribed walking protocols (PWPs) performed on two different days (intra-unit variability). The second hypothesis we tested was that various DG100 units used simultaneously during PWPs show low inter-unit variability when estimating walking distances and speeds. To test and verify these hypotheses, two consecutive experiments were performed.

### Instrumentation

#### GPS recording

During all experiments, one (experiment 1) or four (experiment 2) GlobalSat® DG100 GPS data logger/receiver(s) (GlobalSat Technology Corp., Taiwan, cost approximately $60) were used with an external antenna (AT-65 GPS Active Antenna; GlobalSat Technology Corp., Taiwan, cost approximately $15). Throughout the paper, the abbreviation “DG100” refers to this setup (including the antenna). Technical details about the DG100 have been presented in a recent study on the DG100 accuracy [11]. We refer readers to previous articles for detailed explanation of GPS and EGNOS-enabled GPS specifications [1], [12], [13]. The recording rate for all the devices was 0.5 Hz, as in the previous studies [3], [4], [7]. During all experiments, the DG100 units were placed in a backpack, and the antennas were placed over the backpack. When multiple units were used in experiment 2, antennas were installed next to each other on the top of the backpack.

#### GPS data processing and analysis

After each experiment, data were downloaded from the DG100 using the GlobalSat software utility (Data logger PC utility, version 1.1, 2006). The recorded speeds were analyzed on a personal computer using a spreadsheet (Microsoft® Excel 2000, Microsoft Corporation, USA) with the previously validated specific processing methodology [7]. Using this method both with the DG100 and with another GPS device (Garmin™ GPS 60), we previously shown an accurate detection of walking bouts as well as an accurate estimation of walking distances and speeds [7], [11].

### Elaboration of prescribed walking protocols

For both experiments 1 and 2, a different prescribed walking protocol (PWP) was established for each subject. Each PWP consisted of two consecutive series of walking bouts of 50, 100, 150, 200, 250, 300, 350 and 400 m (total distance for a series = 1800 m; total distance per PWP = 3600 m). For a given PWP, the order of the walking bouts into the first series was randomly predetermined. This order was then replicated for the second series. Throughout all the PWPs, each walking bout was separated by a resting period of ∼30 s.

### Experimental procedure

The PWPs for experiments 1 and 2 were performed on a 400 m synthetic outdoor running track in Angers, France (latitude: 47°28′22″ North; longitude: 0°32′53″ West). The track was identified using blocks placed every 50 m around its circumference. Subjects were asked to perform the first series of the PWP at a “usual” pace and then to perform the second series at a “slow” pace. The task consisted of walking carefully within the interior lane of the athletic track and stopping at the next block upon hearing a whistle blown by the investigator. The whistle was blown when the subject was approximately ten meters from the block. The actual speed of each walking bout was calculated by dividing the distance by the time measured with a stopwatch (Geonaute Trt'L 500, Decathlon Ltd., France).

### Experiment 1: intra-unit variability

#### Objective

Experiment 1 explored the intra-unit variability of the DG100 when estimating outdoor walking distances. Since the walking speed could not be exactly the same between test-retest PWPs, the intra-unit variability was only studied on walking distances estimation.

#### Procedure

Two consecutive PWPs were performed by a sample of ten healthy subjects (M/F: 8/2; 32±5 years, 173±8 cm and 67±9 kg) on two different, non-consecutive days (day A and day B). The ten subjects followed the procedure as described above.

#### Statistical analysis

To investigate the intra-unit variability of the DG100 when estimating walking distances, we calculated i) the typical error in the estimation of the reliability (TEEr) and ii) the coefficient of variation of the reliability (CVr) according to the Hopkins statistical procedure [14]. This statistical procedure compared the GPS distances on day A and day B. To avoid any confusion in regard to the statistical parameters of accuracy computed for experiment 2, the terms TEEr and CVr (“r” for reliability) have been chosen. TEEr corresponds to the standard deviation of the individual difference between DG100 values at day A and day B, and is expressed in absolute value according to the unit of the measured parameter (here in meters for walking distance). CVr looks like the TEEr but it is computed from log-transformed data and is expressed in %, according to the Hopkins statistical procedure [14]. To homogenize and facilitate the methods used to calculate TEEr and CVr, the Hopkins spreadsheet was used [15]. TEEr and CVr are presented with 95% Confidence Interval [95% CI].

### Experiment 2: inter-unit variability

#### Objective

The aim of experiment 2 was to investigate the inter-unit variability in the estimation of walking distances and speeds. For this purpose, we simultaneously used a sample of four DG100 units. Ten healthy volunteers (M/F: 5/5; 21±3 years, 174±3 cm and 68±3 kg) participated to experiment 2. Ten new PWPs were created for experiment 2 (different from those used in experiment 1).

#### Procedure

Subjects followed the same procedure as in experiment 1.

#### Statistical analysis

First, to investigate the inter-unit variability of the DG100 we calculated (i) the typical error in the estimation of accuracy (TEEa) as well as (ii) the coefficient of variation in the estimation of the accuracy (CVa) according to Hopkins statistical procedure [14]. These statistical procedures compared GPS-measured distances and speeds with the actual distances and speeds. Using this procedure, our aim was to report the inter-unit variability in term of “variability in the accuracy” of the estimation for each unit. The term “accuracy” here is only used to differentiate these results from those of experiment 1. Because the TEEa and CVa were calculated for each distance (50, 100, 400 m, etc.), the “validity spreadsheet” could not be used [16]. As suggested by Hopkins (personal communication), the “reliability spreadsheet” was used instead, and the term “√2” was removed from the TEEa and CVa calculation formulas [15]. TEEa and CVa are presented with 95% Confidence Interval [95% CI]. Furthermore, between units comparison of the CVa values was performed using the variance calculated for each CVa and the ratio of the larger variance to the smaller variance for each pairwise comparison. A p-value was calculated from the variance ratio using the F distribution [17]. A p-value<0.05 was considered as statistically significant.

Second, the inter-unit variability was studied by performing unit by unit comparisons (unit 1 *vs.* unit 2, unit 2 *vs.* unit 3,…) and computing TEE and CV.

## Results

No external event interfered with any of the experiments we performed. All subjects closely followed the prescribed instructions for walking and stopping.

### Experiment 1

Experiment 1 investigated the intra-unit variability of the DG100 (test-retest comparison) when estimating the outdoor walking distances using PWPs performed by healthy participants.

**Figure 1:**
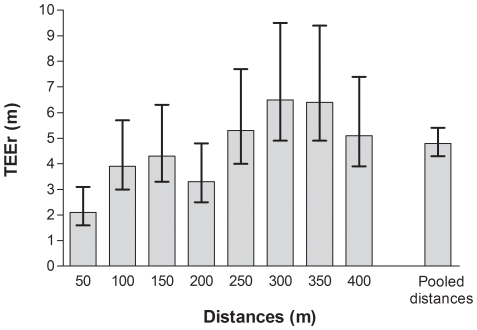
Typical error of the estimate (TEEr, with 95% Confidence Interval) for the reliability of the estimation of walking distances. Note: In the term “TEEr”, “r” means reliability.

**Figure 2:**
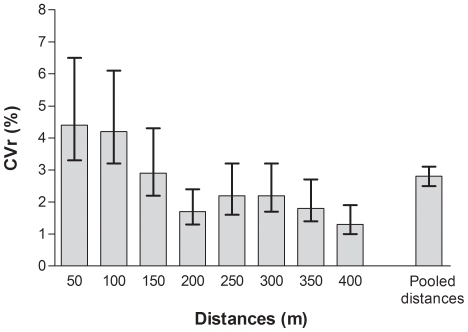
Coefficient of variation (CVr, with 95% Confidence Interval) for the reliability of the estimation of walking distances. Note: In the term “CVr”, “r” means reliability.

No missing sample data, due to possible GPS signal loss, were noticed on the recordings. At day A, mean ± standard deviation of DG100 walking speed was 3.2±0.9 km/h (range 1.4 to 4.5 km/h) for series at “slow” pace, and it was 5.5±0.5 km/h (range 4.7 to 6.3 km/h) for series at “usual” pace. At day B, mean ± standard deviation of DG100 walking speed was 3.2±0.9 km/h (range 1.5 to 4.8 km/h) for series at “slow” pace, and it was 5.5±0.6 km/h (range 4.1 to 6.8 km/h) for series at “usual” pace. Figure 1 and Figure 2 show respectively TEEr and CVr calculations for the intra-unit variability in estimating walking distances. As shown, the TEEr was 4.8 [4.3–5.4] m, and the CVr was 2.8 [2.5–3.1] % for all pooled distances (mean distance walked = 225 m).

### Experiment 2

In experiment 2, we determined the inter-unit variability of DG100 units using simultaneously a sample of four units to estimate walking distances and speeds.

No missing sample data, due to possible GPS signal loss, were noticed on the recordings. Mean ± standard deviation of walking speed for the four DG100 was 3.9±0.6 km/h (range 2.7 to 5.3 km/h) for series at “slow” pace, and it was 5.4±0.5 km/h (range 4.3 to 6.9 km/h) for series at “usual” pace. Table 1 presents, for each DG100 unit, the TEEa and CVa of the estimated walking distances. The TEEa for all pooled distances (mean distance walked = 225 m) among the DG100 units ranged from 6.1 to 10 m. The CVa for all pooled distances among the DG100 units ranged from 2.8 to 3.9%. Although DG100 #1 had the lowest CV (best accuracy) and the DG100 #4 had the highest CV (lowest accuracy), the difference in CVs among the DG100 units did not exceed 1.1%. Expressed in meters and for a mean distance of 225 m, the distance estimation error (TEE) by the DG100 #4 was 2.2 to 3.9 m higher as compared to the three others DG100.

**Table 1:** Inter-unit variability in the accuracy of the estimation of processed walking distances according to the covered distance, for the four DG100 units; the typical error of the estimate (TEEa) and the coefficient of variation (CVa) with 95% Confidence Interval [95% CI] are presented.

	TEEa [95% CI]				
	DG100 units N°				
Distances (m)	1	2	3	4	Whole DG100
50	2.6 [1.9–3.7]	2.4 [1.8–3.5]	2.3 [1.7–3.3]	2.7 [2.1–3.9]	2.5 [2.1–2.9]
100	2.8 [2.1–4.1]	3.2 [2.4–4.7]	3.3 [2.5–4.8]	3.8 [2.9–5.5]	3.2 [2.8–3.8]
150	3.7 [2.8–5.4]	4.0 [3.1–5.9]	4.2 [3.2–6.2]	5.3 [4.0–7.8]	4.4 [3.8–5.2]
200	3.1 [2.4–4.6]	4.8 [3.7–7.1]	5.4 [4.1–7.9]	5.7 [4.3–8.3]	5.0 [4.3–5.9]
250	4.9 [3.7–7.1]	7.8 [5.9–11.4]	6.0 [4.6–8.8]	8.4 [6.4–12.2]	7.2 [6.2–8.5]
300	5.9 [4.5–8.7]	6.3 [4.8–9.2]	6.1 [4.6–8.9]	6.9 [5.2–10.1]	6.6 [5.7–7.8]
350	6.9 [5.3–10.1]	8.4 [6.4–12.3]	8.8 [6.7–12.8]	12.1 [9.2–17.7]	9.4 [8.1–11.2]
400	7.2 [5.4–10.5]	9.3 [7.1–13.7]	10.0 [7.6–14.6]	12.9 [9.8–18.9]	10.3 [8.9–12.2]
All pooled distances (mean distance = 225 m)	6.1 [5.5–6.8]	7.8 [7.0–8.8]	7.6 [6.8–8.5]	10 [9.0–11.3]	8.1 [7.7–8.6]

	CVa [95% CI]				
	DG100 units N°				
Distances (m)	1	2	3	4	Whole DG100
50	5.6 [4.2–8.3]	5.1 [3.9–7.6]	4.8 [3.6–7.0]	5.7 [4.3–8.5]	5.2 [4.5–6.2]
100	2.9 [2.2–4.3]	3.3 [2.5–4.9]	3.5 [2.7–5.2]	4.0 [3.0–5.9]	3.4 [2.9–4.0]
150	2.6 [2.0–3.9]	2.8 [2.2–4.2]	3.0 [2.3–4.4]	3.9 [2.9–5.7]	3.2 [2.7–3.8]
200	1.6 [1.2–2.4]	2.5 [1.9–3.7]	2.9 [2.2–4.2]^1^	3.0 [2.3–4.5]^1^	2.6 [2.3–3.1]
250	2.0 [1.5–3.0]	3.3 [2.5–4.9]	2.5 [1.9–3.7]	3.6 [2.7–5.3]^1^	3.1 [2.6–3.6]
300	2.1 [1.6–3.0]	2.2 [1.7–3.3]	2.1 [1.6–3.1]	2.4 [1.9–3.6]	2.3 [2.0–2.7]
350	2.1 [1.6–3.0]	2.6 [2.0–3.8]	2.7 [2.0–3.9]	3.7 [2.8–5.5]^1^	2.9 [2.5–3.4]
400	1.9 [1.4–2.8]	2.5 [1.9–3.7]	2.6 [2.0–3.9]	3.5 [2.7–5.2]^1^	2.8 [2.4–3.3]
All pooled distances (mean distance = 225 m)	2.8 [2.5–3.2]	3.2 [2.9–3.6]	3.1 [2.8–3.5]	3.9 [3.5–4.4]^1, 2, 3^	3.3 [3.2–3.5]

TEEa is expressed in meters and CVa in percentage. In the terms “TEEa” and “CVa”, “a” means accuracy.

For CVa comparisons and for each DG100 unit, superscript number indicates a significant difference with the corresponding DG100 number. For all comparisons with significant difference, *P*<0.05.

The TEEa for walking speeds was nearly identical for all units and all distances ranging from 0.1 [0.1–0.1] to 0.2 [0.1–0.3] km/h, except for DG100 #4 at 50 m, which had a TEEa of 0.2 [0.1–0.4] km/h. The TEEa was almost identical because the values of walking speed given by the DG100 units contain only one decimal place. The CVa of the estimated walking speeds is reported in Table 2. The CVa for the walking speed estimate of the DG100 #4 was significantly higher than the three other DG100 units. Nevertheless, as for inter-unit estimation of walking distances, the difference in CVs among the DG100 units did not exceed 1.1%.

**Table 2:** Inter-unit variability in the accuracy of the estimation of processed walking speeds according to the covered distance, for the four DG100 units; the coefficient of variation (CVa) with 95% Confidence Interval [95% CI] is presented.

	CVa [95% CI]				
	DG100 units N°				
Distances (m)	1	2	3	4	Whole DG100
50	4.9 [3.7–7.2]	4.7 [3.6–7.0]	3.7 [2.8–5.4]	5.4 [4.1–8.0]	4.6 [4.0–5.5]
100	3.1 [2.4–4.6]	2.8 [2.1–4.1]	3.4 [2.6–5.0]	3.9 [3.0–5.8]	3.3 [2.8–3.9]
150	2.8 [2.1–4.1]	3.7 [2.8–5.5]	3.3 [2.5–4.9]	4.1 [3.1–6.1]	3.6 [3.1–4.2]
200	1.8 [1.3–2.6]	2.6 [1.9–3.7]	3.0 [2.3–4.4]	3.1 [2.3–4.5]^1^	2.7 [2.3–3.2]
250	1.8 [1.4–2.7]	3.1 [2.4–4.6]^1^	2.5 [1.9–3.6]	3.4 [2.6–5.0]^1^	2.9 [2.5–3.5]
300	2.0 [1.6–3.0]	2.3 [1.8–3.4]	2.2 [1.7–3.2]	2.5 [1.9–3.7]	2.4 [2.0–2.8]
350	2.2 [1.7–3.2]	2.6 [2.0–3.8]	2.8 [2.2–4.2]	3.7 [2.8–5.5]^1^	3.0 [2.6–3.5]
400	1.8 [1.4–2.7]	2.4 [1.8–3.6]	2.6 [2.0–3.8]	3.5 [2.6–5.1]^1^	2.7 [2.3–3.2]
All pooled distances (mean distance = 225 m)	2.7 [2.4–3.0]	3.1 [2.8–3.5]	3.0 [2.7–3.3]	3.8 [3.4–4.2]^1, 2, 3^	3.2 [3.1–3.4]

CVa is expressed in percentage. In the term “CVa”, “a” means accuracy.

For CVa comparisons and for each DG100 unit, superscript number indicates a significant difference with the corresponding DG100 number. For all comparisons with significant difference, *P*<0.05.

Finally, Table 3 presents inter-unit comparisons between all DG100 units. Unit-by-unit comparisons of CVs have shown that CVs were ∼3%, both for walking distance and speed inter-unit comparisons. These results were very similar to the results reported in Table 1 and Table 2 regarding the “all pooled distance”.

**Table 3:** Inter-unit comparisons between all DG100 units for walking distances and speeds; the typical error of the estimate (TEE) and the coefficient of variation (CV) with 95% Confidence Interval [95% CI] are presented.

	Walking distance		Walking speed	
DG100 units	TEE [95% CI]	CV [95% CI]	TEE [95% CI]	CV [95% CI]
1 *vs.* 2	6.6 [6.0–7.4]	3.4 [3.0–3.8]	0.1 [0.1–0.1]	3.3 [2.9–3.7]
1 *vs.* 3	5.3 [4.8–5.9]	2.8 [2.5–3.2]	0.1 [0.1–0.1]	2.7 [2.4–3.0]
1 *vs.* 4	7.2 [6.5–8.1]	3.4 [3.1–3.9]	0.1 [0.1–0.2]	3.4 [3.1–3.9]
2 *vs.* 3	6.1 [5.5–6.9]	3.0 [2.7–3.4]	0.1 [0.1–0.1]	3.0 [2.7–3.4]
2 *vs.* 4	7.1 [6.4–7.9]	3.4 [3.1–3.9]	0.2 [0.1–0.2]	3.4 [3.0–3.8]
3 *vs.* 4	6.1 [5.5–6.8]	3.0 [2.7–3.4]	0.1 [0.1–0.1]	2.9 [2.6–3.3]

TEE is expressed in meters for walking distance and in km/h for walking speed. CV is expressed in percentage.

Comparisons were performed from all pooled walking bouts (mean walking distance = 225 m).

## Discussion

This study provides original results about the intra- and inter-variability of a convenient GPS data logger/receiver to study human outdoor walking for health and clinical research studies. There are two major findings in the present study. First, with the DG100 used, we found low intra-unit variability (high reliability) in the estimation of walking distances. Second, among the four units used, we found a very low inter-unit variability in the estimation of walking distances and speeds.

### Intra- and inter-unit variability

A number of recent studies have assessed the intra- and inter-unit variability of different GPS devices [18], [19], [20], [21], [22], [23], [24]. However, the direct comparison of our results with many of these studies is difficult. Indeed, most of these studies used GPS devices designed for sports applications and focused on the measurement of distance and peak speed during high-intensity, intermittent running exercises or during kayak sessions. Few studies analyzed walking locomotion [18], [19], [24]. In addition, most of these studies used relatively expensive devices that analyzed a combination of GPS and accelerometry. These devices use algorithms that retrieve data from the inbuilt, high-frequency accelerometer to correct GPS values, which is expected to improve the accuracy of the GPS devices. Consequently, it is difficult to discuss the “true” accuracy and variability of these GPS devices when estimating speed and distance.

#### Intra-unit variability

The test-retest error when estimating walking distances (intra-unit variability) was within the range of error estimation reported for the DG100 accuracy described in another study [11]. The DG100 compares favorably with the WI SPI elite (GPSports, Canberra, ACT, Australia) used by Gray *et al.* [18]. These authors reported an intra-unit variability of 1.85% and 2.79% for linear and non-linear 200 m walking bouts, respectively. Jennings *et al.* [24] studied the intra-unit variability of two MinimaxX GPS devices (MinimaxX, Team 2.5, Catapult Innovations, Scoresby, Australia) used simultaneously with sampling rates of 1 Hz and 5 Hz, respectively. Interestingly, with the 1 Hz sampling rate, authors reported CVs of 30.8, 20.4 and 7.0% for walking distances of 10, 20 and 40 m, respectively. With the 5 Hz device, authors reported CVs of 23.3, 21.2 and 6.6% for walking distances of 10, 20 and 40 m, respectively. These results indicate that when studying walking locomotion over distances of 20 m or more, there probably is little benefit to using a GPS device with a sampling rate higher than 1 Hz. The CV of 6.6% reported by these authors for the 1 Hz device over 40 m is consistent with the CVs that we found for a 50 m distance using a GPS device sampling at 0.5 Hz. Finally, when using three different types of commercial GPS devices to measure a walking distance of 8800 m, Petersen *et al.* [19] showed an intra-unit variability ranging from 0.3% to 2.6%.

#### Inter-unit variability

One specific interest in conducting the present work was to study the inter-unit variability over a wide range of relatively short distances (50 to 400 m). These distances were expected to cover a range of distances of clinical interest for diseased patients suffering from walking limitations. We found that the four DG100s tested in the present study had a low inter-unit variability. When comparing various WI SPI elite GPS units, Gray *et al.* [18] reported a CV for inter-unit variability (95% CV) of 2.02% and 3.43% over linear and non-linear 200 m walking bouts, respectively. Petersen *et al.* [19] tested the inter-unit variability of several GPS units over a larger walking distance of 1200 m, which automatically reduces the relative variability in their results. These authors reported coefficient of variations from 1.3 up to 1.5%.

Finally, it appears that DG100 #4 consistently showed TEEa and CVa values slightly higher than the other three units. This trend is unlikely to be a result of software differences because the recordings from all four units were processed the same way. The higher TEEa and CVa values observed for DG100 #4 likely stem from hardware differences, even though all four units were bought at the same time from the manufacturer. The differences between units were so slight, however, that they would have very little influence on results that might be obtained in cohort studies, in which multiple units might be needed.

### Potential applications of GPS in health and disease

The GPS technique is an interesting method to objectively estimate free-living walking capacity in patients with walking disability induced by chronic diseases [2], [3], [4], [5], [6], [25]. In addition to walking distance, it can measure usual walking speed and recovery duration between walking bouts. These two parameters have been difficult to examine in laboratory investigations. The GPS technology could also be used to quantify physical activity in healthy and diseased subjects taking into account contextual information (e.g., location) [1]. The question should be asked as to whether the GPS technique is preferable to other types of monitors. High–sampling rate accelerometers are reliable tools for accurately detecting daily physical activities such as walking [26], [27], [28]. Thus, if the only goal is to assess walking and non-walking sequences, accelerometers are accurate. Nevertheless, various limitations have been reported concerning the prediction of speed using accelerometers, including: i) the large inter-subject variability between speed and raw acceleration or accelerometer counts [29], [30]; ii) the complexity of algorithms used to convert raw acceleration to speed and distance [12], [29], [30]; iii) the inability to predict speed when slope changes [12], [29] or when the subject has a pathological gait such as claudication [12]. Further, accelerometers do not allow the user to automatically determine geographic position and the cost of many high-sensitivity accelerometer units limits large cohort studies.

The GPS technique has also some inherent limitations. The major limitation is that the satellite signals are influenced by atmospheric conditions and environmental obstructions, which can produce error in the computed position, speed and distance. In addition, the GPS technique cannot track physical activity indoors where the satellites signals can be lost. Under such circumstances, one potential solution could be to use GPS combined to accelerometry. Indeed, this could be very useful because some patients rarely walk outdoors, and their walking bouts may primarily occur indoors. Various studies have reported interesting data regarding the usefulness of complementing accelerometry measurements with GPS data [31], [32], [33], [34], [35], [36]. This combination of GPS and accelerometry could be extended in a clinical context.

### Study limitation

In the present study, the experiments were all performed in open sky but non controlled open cloud conditions. Open sky conditions refer to low level of obstruction (no buildings and/or dense vegetation). According to the objective of the studies, GPS receivers can be used in very different environments, resulting in different level of obstruction. For instance, the measurement of walking activity performed in a park or in a street would result in different conditions of satellite reception. This would probably result in missing sample data due to GPS signal losses, as well as in a lower accuracy and reliability in the estimation of walking distances and speeds. To date, no study has addressed this issue during (walking) locomotion. Open cloud conditions refer to atmospheric conditions that can influence satellite signals. For instance, cloud cover may have adverse effects on GPS signal, which can lead to the decrease of GPS accuracy and reliability. In the present study, rainy days were avoided but we could not control cloud cover. We advocate that this had little influence on the intra-units variability (day-to-day variability), and no significant influence on the inter-units variability since GPS units were used simultaneously.

Another limitation is that we did not study the intra-unit reliability of the DG100 in the estimation of walking speed. Although walking speed was not controlled, subjects were asked to perform the first series of the PWP at a “usual” pace and then to perform the second series at a “slow” pace. Using this procedure, we expected to cover a large range of walking speeds. For instance, in experiment 2 the different walking speeds covered a range of 2.7 to 6.9 km/h. Interestingly, in a previous work on 24 peripheral artery disease patients [3], we reported a median (25^th^–75^th^ percentiles) GPS walking speed of 3.6 (3.4–4.2) km/h, which is within the range of the walking speeds reported in the present study. This strengthens the external validity of our results in view of clinical applications in diseased subjects.

To the best of our knowledge, no study has been published specifically on the effect of walking speed on both GPS accuracy and reliability during walking. In the present study, the subjects were asked to perform PWP at low and usual pace, but we did not analyze the potential effect of walking speed on the intra- and inter-unit variability. Such analyses would require the same number of walking bouts within each interval of walking speed assessed (*i.e.* 1 to 2 km/h, 2 to 3 km/h…) and for each walking distance (50, 100… 400 m). This was not the case in the present study. Future experiments need to address this issue, particularly for very low walking speeds (below 3 km/h), as it can be sometimes encountered in elderly or poorly fit subjects.

### Conclusion

The present work, in addition to the recently published study [11], indicates that the DG100 produces sufficient accuracy and reliability to study human outdoor walking in open sky conditions. This could facilitate future works focusing on outdoor walking in perspective of health and clinical research studies. We advocate that GPS data logger/receivers should be validated (*i.e.*, tested for accuracy and reliability) before use. Very low-cost GPS units can facilitate the evaluation of human outdoor walking in multicenter or large-cohort studies on health and disease. However, future studies should focus on the effect of very low walking speed on both GPS accuracy and reliability.
